# Supplementary material for: A SNaPshot Assay for Determination of the Mannose-Binding Lectin Gene Variants and an Algorithm for Calculation of Haplogenotype Combinations
Source: Diagnostics (Basel). 2021 Feb 13;11(2):301. doi: 10.3390/diagnostics11020301 (PMC7918147; doi:10.3390/diagnostics11020301)
Supplement: Supplementary file 1 [file diagnostics-11-00301-s001.pdf]

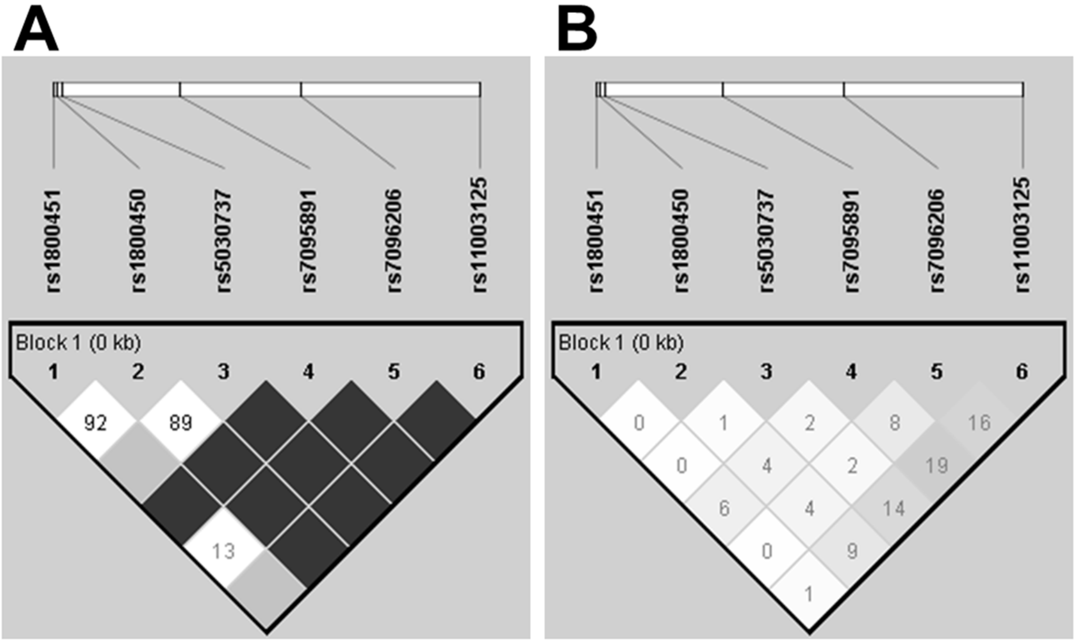

Figure S1. Linkage disequilibrium (LD) maps for 6 single nucleotide polymorphisms in the mannose-binding lectin gene (*MBL2*): (**A**) The pairwise LD display based on D'. Black squares represent D' = 1, grey squares represent D' = 1 with the logarithm of the odds (LOD) < 2; the white number-containing squares represent the value of D' in percent with LOD < 2; (**B**) The pairwise LD display based on r^2. Shades of grey represent the strength of LD, the number represents the value of r^2 in percent. All SNPs belong to the LD block marked by a black triangle (identified using the Solid spine of the LD method).
